# Ras enhances TGF-β signaling by decreasing cellular protein levels of its type II receptor negative regulator SPSB1

**DOI:** 10.1186/s12964-018-0223-4

**Published:** 2018-03-13

**Authors:** Sheng Liu, Josephine Iaria, Richard J. Simpson, Hong-Jian Zhu

**Affiliations:** 1 Department of Surgery (RMH), The University of Melbourne, The Royal Melbourne Hospital, Parkville, VIC 3010 Australia; 2 Department of Biochemistry, La Trobe Institute for Molecular Science, La Trobe University, Bundoora, VIC Australia

**Keywords:** Ras, SPSB1, TGF-β signaling

## Abstract

**Background:**

Transformation by oncogene Ras overcomes TGF-β mediated growth inhibition in epithelial cells. However, it cooperates with each other to mediate epithelial to mesenchymal transition (EMT). The mechanism of how these two pathways interact with each other is controversial.

**Methods:**

Molecular techniques were used to engineer expression plasmids for Ras, SPRY, TGF-β receptors, type I and II and ubiquitin. Immunoprecipitation and western blots were employed to determine protein-protein interactions, preotein levels, protein phosphorylation while immunofluorecesent staining for molecular co-localization. TGF-β signalling activities is also determined by its luciferase reporter assay. Trans-well assays were used to measure cell migration and invasion.

**Results:**

Ras interacts with the SPSB1’s SPRY domain to enhance TGF-β signaling. Ras interacts and colocalizes with the TGF-β type II receptor’s (TβRII) negative regulator SPSB1 on the cell membrane, consequently promoting SPSB1 protein degradation via enhanced mono- and di-ubiquitination. Reduced SPSB1 levels result in the stablization of TβRII, in turn the increase of receptor levels significantly enhance Smad2/3 phosphorylation and signaling. Importantly, forced expression of SPSB1 in Ras transformed cells suppresses TGF-β signaling and its mediated migration and invasion.

**Conclusion:**

Ras positively cooperates with TGF-β signaling by reducing the cellular protein levels of TβRII negative regualtor SPSB1.

**Electronic supplementary material:**

The online version of this article (10.1186/s12964-018-0223-4) contains supplementary material, which is available to authorized users.

## Background

TGF-β regulates a plethora of cellular processes including cell proliferation, differentiation, migration, organization and death [1]. As one of the most potent inhibitors of normal cell growth, the loss of growth inhibitory responses to TGF-β is often observed in cancer cells [2, 3]. It is widely accepted that TGF-β is a tumor suppressor, given the frequent occurrence of many types of tumors in mice with disruptions of TGF-β signaling components by gene targeting and many types of human cancers containing loss-of-function mutation of TGF-β signaling components [4]. In spite of the tumor suppressor activity of TGF-β, the majority of human tumors have not suffered loss-of function of TGF-β signaling components [5]. Tumor cells, particularly advanced tumor cells, often show increased production of TGF-β while they are insensitive to TGF-β induced growth inhibition [6]. TGF-β acting as an important tumor promoter, particularly at late stages of tumor development, is evidenced by using murine animal models and human cellular systems [7–10], in which TGF-β signaling components are required for tumor invasion in vitro and metastasis in vivo. Clinically, there is a substantial body of evidence that excess TGF-β production is associated with poor prognosis in many types of human tumors [5]. Thus, TGF-β acts as a tumor suppressor in early tumor development, but promotes tumor invasion and metastasis during late stages of tumor progression [11].

Ras proteins are small GTPases that act as molecular switches by cycling between inactive GDP-bound and active GTP-bound states. It functions as a transducer of the cell signals from the membrane receptor to the intracellular pathway that controls cell proliferation, differentiation, and survival [12]. Constitutive active mutations of Ras are frequently expressed in human cancers— ~ 20 to 30% of all human tumors contain one of the mutated Ras genes, especially in pancreas, thyroid and colon carcinomas (90, 60 and 45% respectively) [13, 14]. Ras plays an important role in tumor initiation as well as in tumor maintenance [15]. Many carcinomas carrying the activated Ras proteins have undergone EMT [16, 17]. It is known that Ras downstream effecter pathway Ras-Raf-MAPK is essential mediating EMT [10, 18]. On the other hand, activation of another Ras downstream effecter pathway PI3K/Akt enhances tumor cell growth and mediates protection from TGF-β induced apoptosis [19, 20].

TGF-β and Ras signaling are two of the most important molecular pathways mediating the fundamental cellular process, namely EMT, involved in tumor metastasis [21, 22]. Depending on the cellular contexts, Ras signaling antagonizes TGF-β-induced growth arrest and apoptosis [23] by suppressing the TGF-β-Smad signaling [24]. It was reported that Ras, acting through Mek1 and Erk kinases, induced the phosphorylation of Smad2/3 at a cluster of Ser/Thr-Pro sites in the linker region [24]. The Ras-induced phosphorylation in the linker region prevents the accumulation of Smad2/3 in the nucleus. Prolonged activation of Raf/MAPK pathway in MDCK cells significantly reduces Smad3 levels independently of TGF-β stimulation [25]. Recently, Ras has been shown to induce the down-regulation of TβRII [26]. Induced expression of mutant Ras activates MAPK pathway which leads to the recruitment of histone deacetylase (HDAC). HDAC suppresses the TβRII promoter region (− 127/− 75) and consequently results in the down-regulation of TβRII in lung cancer cells [26]. In contrast, Ras signaling was shown to up-regulate TGF-β production [27], enhancing endogenous TGF-β signaling [10]. During advanced stage of tumour development, Ras signaling often positively cooperates with TGF-β signaling. Activation of the Ras-MAPK signaling often results in autocrine TGF-β signaling which is critical in EMT maintenance [9, 19, 27–29]. It has been shown that metastasis is driven by sequential elevation of H-Ras and Smad2 levels [10]. During tumour progression from keratinocyte towards squamous carcinoma then to invasive spindle cell carcinoma, TGF-β signaling activity was dramatically increased and the constitutively activated Smad2 was observed in invasive spindle tumour cells only. Activated H-Ras over-expression in squamous carcinoma cells demonstrated that oncogenic Ras stimulated TGF-β-induced transcription and enhanced TGF-β-induced phosphorylated Smad2 levels [10]. However, how Ras positively regulates the TGF-β signaling is unclear. The mechanisms of cross-talk between the Ras and TGF-β signaling are being investigated in a number of cell lines, with controversial results [30].

Recently, we have identified SPSB1 (SPRY domain-containing a SOCS box protein 1) as a novel negative regulator of the TGF-β signaling pathway [31]. The SPSB1 gene expression is induced by TGF-β and it feeds back to negatively regulate the TGF-β signaling pathway. Interestingly, SPSB1 has also been reported to positively regulate the c-MET-Ras-MAPK signaling [32]. We investigate whether SPSB1 bridges the Ras and TGF-β signaling. This study describes a new mechanism of how Ras up-regulates the TGF-β signaling: Ras interacts with the newly identified TβRII negative regulator SPSB1 and causes its degradation via ubiquitination. This leads to the enhanced TβRII levels and consequently increased TGF-β signaling activity. This is the first to report that Ras is directly targeting TGF-β signaling regulatory components to enhance its signaling activity.

## Experimental procedures

### Antibodies and reagents

The mouse anti-FLAG (M2) and anti-Actin monoclonal antibodies were obtained from Sigma-Aldrich (St Louis, MO). Mouse monoclonal anti-MYC and anti-Ras (detects endogenous Ras) antibodies were generated in house. Rabbit polyclonal anti-TβRI, anti-TβRII and mouse monoclonal anti-H-Ras antibodies were obtained from Santa Cruz Biotechnology (Santa Cruz, CA). Rabbit polyclonal anti-FLAG antibody was obtained from ABR (Affinity BioReagents, Golden, CO). Rabbit polyclonal anti-phospho-Smad2 antibody was kindly provided by Prof Peter ten Dijke (Leiden University Medical Center, Netherlands). Mouse monoclonal anti-Smad2 antibody was obtained from BD Transduction Laboratories (Rockville, MD). Goat anti-mouse IgG HRP conjugated secondary antibody, Goat anti-rabbit IgG HRP conjugated secondary antibody were obtained from Bio-Rad (Bio-Rad Laboratories, Gladesville, N.S.W., Australia). The anti-mouse Alexa^488^ and Alexa^546^-conjugated secondary antibodies were from Invitrogen (Invitrogen Corp., Mulgrave, Australia). Human recombinant TGF-β1 was obtained from R&D Systems (Minneapolis, MN). Doxycycline and Cycloheximide were purchased from Sigma-Aldrich, while MG132 was obtained from Merck (Merck, Darmstadt, Germany).

### DNA constructs and primers

FLAG-TβRI, HA-TβRII and v-Ha-Ras were cloned into pcDNA3 mammalian cell expression vector as described previously [33, 34]. v-Ha-Ras(N85A), v-Ha-Ras(N86A) and v-Ha-Ras(D120A, R124A) were generated based on v-Ha-Ras using Quick Change® II XL Site-Directed Mutagenesis Kit (Agilent Technologies, Santa Clara, CA) according to the manufacturer’s recommendations. The following pimers were used in the PCR reaction: v-Ha-Ras N85A forward: TGTGTATTTGCCATCGCCAACACCAAGTCCTT. v-Ha-Ras N85A reverse: AAGGACTTGGTGTTGGCGATGGCAAATACACA. v-Ha-Ras N86A forward: GTATTTGCCATCAACGCCACCAAGTCCTTTGA. v-Ha-Ras N86A reverse: TCAAAGGACTTGGTGGCGTTGATGGCAAATAC. v-Ha-Ras D120A, R124A forwards: TGGGCAACAAGTGTGCACTGGCCGCTGCCACTGTTGAGTCTC. v-Ha-Ras D120A, R124A reverse: GAGACTCAACAGTGGCAGCGGCCAGTGCACACTTGTTGCCCA. The sequence of all newly generated v-Ha-Ras mutants were confirmed by direct DNA sequencing. FLAG/MYC-SPSB1, FLAG/MYC-SPSB1∆, MYC-SPSB1(Y129A) and MYC-SPSB1(T160A, Y161A) were all cloned into the *pEF-BOS* mammalian cell expression vector [35].

### Cell lines, cell culture and treatments

The human embryonic kidney cell line HEK-293 T (293 T), the Madin Darby Canine Kidney (MDCK) cell line, the v-Ha-Ras stable transformed MDCK (21D1) cell line have all been previously described [32, 34]. To generate the doxycycline inducible SPSB1 cell line in 21D1 cells, a tetracycline-inducible vector, pTRE was utilized [36]. Briefly, pTRE-FLAG-SPSB1 and *pEFpurop-Tet-on* [36] were co-transfected into 21D1 cells by using FuGENE HD transfection reagent (Roche, Basel, Switzerland) following the manufacturer’s instructions and selected for using puromycin (Roche, Basel, Switzerland). To generate the doxycycline inducible v-Ha-Ras cell line in MDCK cells, pTRE-v-Ha-Ras and *pEFpurop-Tet-on* were co-transfected into MDCK cells by using FuGENE 6 transfection reagent (Roche, Basel, Switzerland) following the manufacturer’s instructions and selected for using puromycin (Roche, Basel, Switzerland). All positive clones were selected by Western blot analysis using FLAG antibody (Sigma-Aldrich) or Ras antibody (In house made). All cells were maintained in Dulbecco’s Modified Eagle’s Medium contained 10% foetal bovine serum (FBS) (DKSH, Hallam, Victoria, Australia), 2 mM glutamine, 100 U/ml penicillin and 100 μg/ml streptoMYCin (Invitrogen).

### Luciferase assays

Cells were transiently transfected with firefly luciferase (luc) construct *pCAGA*_*12*_*-luc* [37], along with other DNA constructs as indicated using FuGENE HD transfection kit for 293 T cells and METAFECTENE PRO (Biontex Laboratories, San Diego, CA) for all other cells. Twenty-four hours after transfection, cells were stimulated with ± TGF-β at indicated concentration in medium containing 10% FCS for a further 24 h. Thereafter, cells were lysed and assessed for luciferase activity using the Luciferase Reporter Assay Kit (Promega Corp, Madison, WI) following the manufacturers’ instructions.

### Immunoprecipitation and immunoblotting

After transfection, cells were lysed in lysis buffer (50 mM Tris, 150 mM NaCl, 1% Triton-X-100, 50 mM NaF, 2 mM MgCl_2_, 1 mM Na_3_VO_4_, 25 μg/ml leupeptin and 25 μg/ml aprotinin) and cell lysates were subjected to immunoprecipitation with appropriate antibody conjugated sepharose protein G bead or anti-FLAG beads (Sigma-Aldrich) for 4 h. Immunoprecipitates were washed three times with ice-cold PBS containing 0.5% Tween-20 and immunoprecipitated proteins were separated by SDS-PAGE (Invitrogen) and blotted onto nitrocellulose membrane and probed with the indicated primary antibodies. The signal was visualized using the ECL chemoluminescence detection kit (GE Healthcare, Rydelmere, N.S.W., Australia) following incubation with appropriate secondary antibodies.

### Qualitative analysis for protein half life

The intensity of the bands in western blot images was measured using image J. Rectangular selection tool was used to select the area where the bands were located (the intensity of bands that were used to calculate the half-life of the protein was measured together in one selected area). The gaps between each band were used as relative background. The intensity of each band was measured 3 times by selecting three different gap intensities as the relative background (background intensity selected at low, medium and high). Protein stability curves were generated by smoothly joining the intensity values of each set of bands in the Y-axis, with their corresponding treatment times in the X-axis using Microsoft excel. Half-life was determined as the time at which protein band intensities were 50% of the starting level (time 0). The value of half-life shown in the results is the mean of the three estimated half-life values for each four bands. The results are shown as the mean of estimated half-life values +/− SD.

### Immunofluorescence staining and confocal microscopy

After transfection with appropriate DNA constructs using FuGENE HD for 48 h, cells were washed once in pre-heated 37°C PBS and fixed with 3.7% formaldehyde (Sigma) in PBS for 7 min. Following two PBS washes, cells were permeabilized with 0.2% Triton-X-100 (Merck) in PBS for another 7 min. Cells were then wash 3 times with PBS and blocked with PBS containing 5% BSA for 1 h at room temperature. Following another 3 washes in PBS, cells were stained with relevant primary antibody (diluted in PBS containing 2% BSA) for 1 h at room temperature and washed in PBS 3 more times. Visualisation was achieved with either Alexa^546^ or Alexa^488^-conjugated secondary antibody using the Nikon TE2000-E & C1 Confocal Microscope with a Nikon 60X water immerged lens. Nikon confocal EZ-C1 v.1.4 was used to collate images.

### In vitro scratch assay and fluorescence microscopy

21D1 cells were transfected with indicated DNA constructs and seeded onto 12-well culture plate until 100% confluent. Forty-eight hours post-transfection, scratches were created using a P1000 pipette tip to scratch a straight line on the culture plate. The culture medium was replaced with fresh medium to remove detached cells. Phase-contrast and fluorescence images were acquired at 0 and 24 h post-scratch using an inverted microscope (IX50, Olympus) equipped with a CCD camera (Model 11.3, Diagnostics instruments, MI), and SPOT advanced imaging software (v4.0.4) was used to acquire and process images.

### In vitro invasion and migration assay

21D1 cells were transfected with indicated DNA constructs in a 6-well plate. Forty-eight hours post-transfection, 21D1 (20,000 cells/chamber) cells were resuspended in serum free DMEM and seeded in the top chamber of a 70 μl solidified matrigel (BD Biosciences, 1:1 mixed with DMEM) coated, 8 μm, polycarbonate membrane transwell insert (Corning Incorporated, Corning NY). Serum free DMEM ±2 ng/ml of TGF-β was added to the bottom chamber. Cells were then incubated for 24 h at 37 °C with 10% CO_2_. Thereafter, cells that invaded through the coated matrigel and migrated to the other side of the membrane of the transwell insert were fixed with 3.7% formaldehyde (Sigma) for 7 min. Cells were then washed and stained with Hoechst for 5 min. Any remaining cells in the top chamber of the transwell insert were removed by using a cotton swab. Only cells in the bottom side of the transwell insert were counted. Fluorescent images were taken in three random fields (20×) per insert. Assays were performed in triplicate.

### Statistics

All statistical analyses were performed using a two-tail Students’ T-test (*P* < 0.05 indicating statistical significance).

## Results

### Ras reduces SPSB1 expression levels

**Fig. 1:**
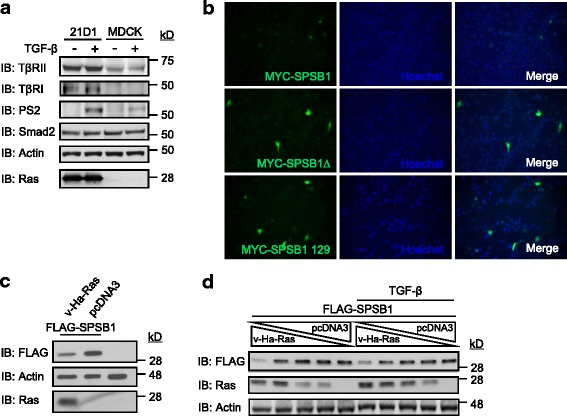
Oncogenic Ras reduces SPSB1 expression level. Cultured MDCK and 21D1 cells (**a**) were treated ± TGF-β (2 ng/ml) for 15 min and then lysed. Whole cell lysates were examined for indicated proteins by immunoblotting (IB). 21D1 cells (**b**) were transfected with indicated DNA constructs (0.2 μg/well each) in a 12 well plate for 48 h. Fixed cells were then immunostained with mouse anti-MYC followed by Alexa488-conjugated secondary anti-mouse IgG. Cell nuclei was stained with Hoechst dye. The expression of SPSB1/SPSB1 mutants (green) was analyzed by fluorescent microscope (magnification = 40×). 293 T cells (**c**) were transfected with indicated DNA constructs for 48 h. Cells in (**d**) were transfected with the FLAG-SPSB1 (0.5 μg/well) and decreasing concentration of v-Ha-Ras (0.5, 0.25, 0.125, 0.0625, 0 μg/well), the total amount of DNA per transfection was kept the same by compensating with pcDNA3 vector. 24 h later, cells were treated with ± TGF-β (2 ng/ml) for a further 24 h. Whole cell lysates were examined for indicated proteins by immunoblotting (IB). In all case, each experiment was repeated, with representative results shown

We have previously generated a mesenchymal cellular model 21D1 by transforming MDCK cells using v-Ha-Ras [34, 38]. While activation of both Ras and TGF-β signalings are required for the maintenance of 21D1 cells mesenchymal phenotype, it is interesting that Smad2 phosphorylation levels are increased in 21D1 cells in comparision to the partental epithelial MDCK cells (Fig. 1a). Further examination revealed that both the TGF-β type I and type II receptors (TβRI and TβRII) levels are elevated in 21D1 cells (Fig. 1a, similar results are obtained in another clone 21F3, results not shown). The SPRY domain containing a SOCS box protein 1 (SPSB1, also known as SSB1), is a newly discovered TGF-β signaling negative regulator by targeting TβRII for degradation. We investigate whether SPSB1 has the ability to reduce Ras caused elevation of TβRII. Surprisingly, while there was no difficulty to over-express SPSB1 protein in the parental MDCK cells [31], expression of SPSB1 in 21D1 cells was very difficult as demonstrated in Fig. 1b that there were hardly any positive immunofluorescence staining. In contrast, the SOCS box deletion mutant (SPSB1∆) or the interaction defective SPRY domain mutant (SPSB1(Y129A)) of SPSB1 was readily to be detected by immunofluorescence staining after transfection (Fig. 1b). Those observations suggest that Ras may paly a role in suppressing the expression of SPSB1.

To investigate the effect of Ras on the expression of SPSB1, we co-transfected FLAG-SPSB1 with v-Ha-Ras in 293 T cells. As shown in lane 2 of Fig. 1c, SPSB1 was readily expressed in the absence of Ras. However, the expression level of SPSB1 was reduced in the presence of Ras (lane 1, Fig. 1c), suggesting that Ras suppresses SPSB1 expression directly. Indeed, when Ras expression levels were progressively reduced, the expression levels of SPSB1 were progressively restored with or without TGF-β treatment (Fig. 1d), confirming the notion that Ras reduces SPSB1 expression level. Furthermore, EGF-induced activation of endogenous Ras has no effect on the degradation rate of SPSB1 (Additional file 1: Figure S1), suggesting a Ras activation independent mechanism.

### Ras interacts with SPSB1 through the SPRY domain

**Fig. 2:**
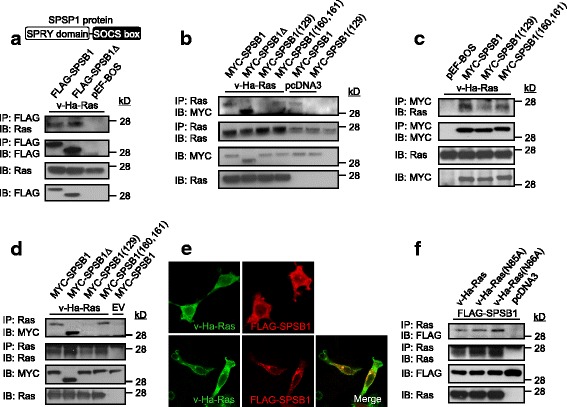
Ras interacts with the SPRY domain of SPSB1. 293 T cells (**a**, **b**, **c**, **d**, **f**) were transfected with indicated DNA constructs (0.5 μg/well each). 48 h later, cell lysates were immunoprecipitated (IP) with anti-Ras antibody (**b**, **d**, **f**) or anti-MYC antibody (**c**) conjugated with protein G beads or anti-FLAG beads (**a**). Both whole cell lysates and immunoprecipatates were examined for indicated proteins by immunoblotting (IB). 293 T cells (**e**) were transfected with v-Ha-Ras or FLAG-SPSB1 singly (top two images) or co-transfected with v-Ha-Ras and FLAG-SPSB1 (bottom images) for 48 h. Fixed cells were then immunostained with rabbit anti-FLAG followed by Alexa^546^-conjugated secondary anti-rabbit IgG and/or mouse anti-Ras followed by Alexa^488^-conjugated secondary anti-mouse IgG as indicated. The sub-cellular localization of SPSB1 (red) and v-Ha-Ras (green) was analyzed by confocal microscope (magnification = 60×). Co-localization of merged images appears as yellow. All experiments were repeated three times, with representative results shown. A figure illustration of SPSB1 protein is shown in **a**

We next examined its interaction with SPSB1 using 293 T cells. Western blot analysis of immunoprecipitates of FLAG-tagged SPSB1 showed the existence of Ras (Fig. 2a), indicating complex formation between Ras and SPSB1. Like SPSB1 interaction with TβRII [31], the SOCS box is not required for its Ras interaction since the deletion mutant (SPSB1∆) formed a complex as efficient as the wild type (Fig. 2a). Those results indicate that the interaction is mediated through the SPRY domain of SBSP1. Reversed immunoprecipitation using Ras antibody confirmed Ras interaction with SPSB1 (Fig. 2b). That mutation at position 129 of SPSB1 disrupted its abililty to form complex with Ras (Fig. 2b) identifies the critical role of Y129 in the SPRY domain for the interaction with Ras. It is noted that this Ras antibody also pulled-down the endogenous Ras which formed immuno-complex with over-expressed SPSB1 (Fig. 2b) suggesting endogenous Ras interaction with SPSB1. Furthermore, this interaction is not significantly affected by EGF stimulation (Additional file 1: Figure S2), suggesting Ras-activation independent interaction. Using another Ras antibody which did not pull-down endogenous Ras, there was no SPSB1 observed in the immuno-complex without over-expression of Ras (Fig. 2d), confirming the specificity of the interaction. However, the double mutant in the SPRY domain (SPSB1 T160A, Y161A) did not appear to interrupt the Ras-SPSB1 interaction (Fig. 2b & d). The differential effect of Y129 from T160/Y161 in interaction with Ras was also confirmed by targeting SPSB1 for immunoprecipitation using MYC-tagged constructs (Fig. 2c). To confirm that the Ras-SPSB1 interaction was not an artifact of cell lysis, we performed co-immunofluorescence staining. When expressed alone in 293 T cells, SPSB1 was diffusely localized in the cytoplasm region (Fig. 2e, top panel). Ras had a similar localization pattern as SPSB1 except it could also be detected on the cell membrane when expressed alone (Fig. 2e, top panel). Interestingly, in the presence of Ras, a substantial portion of SPSB1 was found redistributed to the cell surface (Fig. 2e, bottom panel), co-localizing with Ras on the cell membrane and in the endosome like vesicles. This result is consistent with the co-immunoprecipitation data demonstrating Ras-SPSB1 interaction. Taking together, Ras interacts with the SPRY domain of SPSB1, supporting the previous finding that SPRY domain is functioning as a protein-protein interaction interface [35, 39]. The EGF independent endogenous Ras interaction with SPSB1 observed in Fig. 2b & Additional file 1: Figure S2 suggests that the active GTP-bound Ras may not be required for the Ras-SPSB1 interaction since the endogenous Ras are largely not active in GDP-bound form.

### Ras N85 and N86 are not responsible for its interaction with SPSB1

We and others have previously shown that SPSB1 could recognize D-I-N-N-N-X or similar sequence motifs present in multiple target proteins [31, 39]. In order to further investigate the Ras-SPSB1 interaction, we searched for similar motifs in Ras. Our sequence alignment shows Ras contains a stretch of Ile^84^-Asn-Asn-Thr-Lys^88^ (I-N-N-T-K) in the middle region of the protein. Based on the importance of the asparagine residues in other SPSB1 interacting proteins (TβRII, Par4, VASA and iNOS) [31, 35, 40–42], we generated two mutant v-Ha-Ras DNA constructs, one containing a N85A substitution and the other N86A. Neither mutation disrupted the function of the v-Ha-Ras in mediating Erk1/2 phosphorylation (Additional file 1: Figure S3), indicating correct folding of the expressed mutants. Anti-Ras immuoprecipitation showed that the SPSB1 was co-precipitated with Ras(N85A) and Ras(N86A) at similar potency as the wild-type Ras (Fig. 2f). This non-disruption of interaction was confirmed by performing a reciprocal immunoprecipitation of SPSB1 (Additional file 1: Figure S4). This suggests that although Ras protein contains a similar motif as D-I-N-N-N-X, the asparagine residues are unlikely to be involved in the Ras-SPSB1 interaction.

To further investigate the Ras-SPSB1 interaction, an alternative strategy was employed. Infact, Ras is not the first protein identified to interact with SPSB1 without carrying the D-I-N-N-N-X motif. The HGF receptor c-Met has been reported to interact with SPSB1, however, sequence alignment suggests that c-Met does not contain any sequence similar to the D-I-N-N-N-X motif [32]. We hypothesize that Ras may share a similar interaction motif with c-Met to interact with SPSB1. Sequence alignment between Ras and c-Met identified an identical short stretch of Asp^120^-Leu-Ala-Ala-Arg^124^ (D-L-A-A-R). The charged residues in the D-L-A-A-R sequence were mutated into alanine (v-Ha-Ras(D120A, R124A)). Surprisingly, the mutant protein expression level was low (Additional file 1: Figure S3), however, the double amino acid substitutions did not impair the ability of oncogenic Ras to mediate Erk1/2 phosphorylation (Additional file 1: Figure S3). Interestingly, while Ras(D120A, R124A) was expressed at much lower levels than its wild-type conterpart, a significantly more Ras(D120A, R124A) was detected in the anti-MYC immunoprecipitates (Additional file 1: Figure S5). Furthermore, this interaction between Ras(D120A, R124A) and SPSB1 was confirmed by performing a reciprocal immunoprecipitation of Ras (Additional file 1: Figure S6). As such, the D-L-A-A-R sequence in Ras may be involved for the Ras-SPSB1 interaction.

### Ras down-regulates the expression levels of SPSB1 by enhancing SPSB1 ubiquitination

**Fig. 3:**
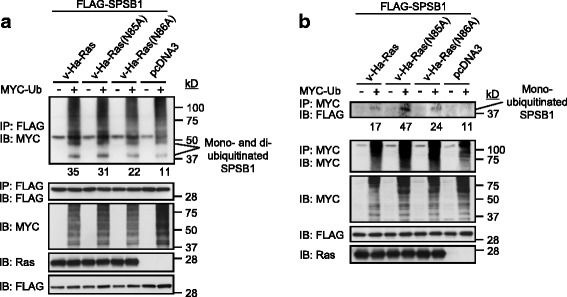
Oncogenic Ras enhances the ubiquitination levels of SPSB1. 293 T cells (**a**, **b**) were transfected with indicated FLAG-SPSB1 construct together with v-Ha-Ras/v-Ha-Ras mutants or pcDNA3 control vector ± MYC-ubiquitin. Cells were lysed 48 h post-transfection. Cell lysates were immunoprecipitated (IP) with FLAG beads (**a**) or anti-MYC antibody conjugated with protein G beads (**b**). Both whole cell lysates and immunoprecipatates were examined for indicated proteins by immunoblotting (IB). Relative densities of mono-ubiquitinated bands are list below the top panels. Results are representative of experiments repeated at least once

How does Ras interaction with SPSB1 suppresses the expression of SPSB1? Reduced protein expression can be caused by enhanced protein ubiquitination and hence degradation. FLAG-SPSB1 was co-transfected with v-Ha-Ras/mutants/pcDNA3 vector as indicated with or without MYC-ubiquitin in 293 T cells. As shown in the last lane of Fig. 3a, when SPSB1 was expressed alone with ubiquitin, mono-ubiquitinated, di-ubiquitinated and poly-ubiquitinated SPSB1 could be detected in the anti-FLAG immunoprecipitates. This is consistent with our early result that SPSB1 itself is a target for ubiquitination [31]. In contrast, when oncogenic Ras was co-expressed, although we did not observed any significant changes in levels of poly-ubiquitinated SPSB1, detectable enhancement of the mono-ubiquitinated (quantitated), di-ubiquitinated and possibly tri-ubiquitinated SPSB1 levels was observed (Fig. 3a). To confirm this notion, a reciprocal immunoprecipitation of MYC for ubiquitin was performed. In the MYC immunoprecipitation, mono-ubiquitinated SPSB1 were only detected in the presence of oncogenic Ras (Fig. 3b).

Since early result indicates that oncogenic Ras does not require the I-N-N-T-K sequence for its interaction with SPSB1, Ras(N85A) and Ras(N86A)’s ability to enhance SPSB1 ubiquitination were examined. As we expected, both Ras(N85A) and Ras(N86A) enhanced the levels of mono-ubiquitinated, di-ubiquitinated and possibly tri- ubiquitinated SPSB1 (Fig. 3a). Again, the result was confirmed by reciprocal immunoprecipitation of MYC for ubiquitin (Fig. 3b). Collectively, these results suggest that oncogenic Ras has the ability to induce the mono-, di- and possibly tri-ubiquitination of SPSB1.

**Fig. 4:**
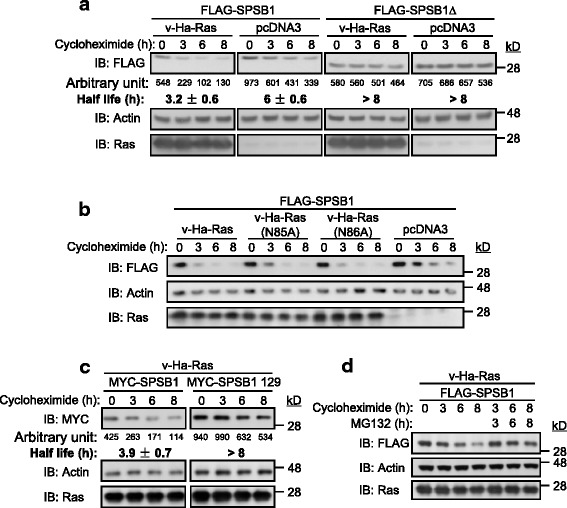
Oncogenic Ras increases the degradation rate of SPSB1. 293 T cells (**a**, **b**, **c**, **d**) were co-transfected with various SPSB1 and Ras/control construct as indicated. 36 h later, cells were exposed to cycloheximide (20 μg/ml) for indicated periods. Cells in (**d)** were co-treated with MG132 (25 μM) for indicated periods. Whole cell lysates were then examined for indicated proteins by immunoblotting (IB). Relative intensity of each SPSB1/SPSB1 mutant band (**a** and **c**) was qualitatively measured using ImageJ, the number under each band indicated its corresponding relative intensity (Arbitrary Units). The half life ± S.D. (*n* = 3 technical replicates) for SPSB1/SPSB1 mutant degradation is shown underneath. In all case, each experiment was repeated at least once, one representing result is showing

Normally, increased ubiquitination of a protein decreases its stability, and consequently its half-life. To measure the protein half-life, the protein synthesis inhibitor cycloheximide was used. Consistent with our previous result [31], the degradation of SPSB1 was observed during the 8 h of cycloheximide treatment when it was expressed alone (Fig. 4a). In the presence of oncogenic Ras, not only was the steady state levels of SPSB1 reduced, but also the protein degradation rate of SPSB1 was increased (Fig. 4a). The SPSB1 protein half-life was calculated by quantitatively measuring the intensity of each band using ImageJ. The expression of oncogenic Ras reduced the protein half-life of SPSB1 from ~ 6 h to ~ 3.2 h (Fig. 4a). In contrast, oncogenic Ras failed to promote the protein degradation of SPSB1(Y129A) (SPSB1 mutant that disrupted the Ras-SPSB1 interaction, Fig. 4c). Furthermore, the expression of oncogenic Ras did not alter the protein stability of SPSB1∆ (Fig. 4a) which was consistent with our early results that the SOCS box was required for the degradation of SPSB1 [31]. Since Ras(N85A) and Ras(N86A) enhance the ubiquitination of SPSB1, they should also enhance the protein degradation of SPSB1. As expected, similar to the wild-type oncogenic Ras, the presence of Ras(N85A) and Ras(N86A) both resulted in observable enhancement of protein degradation of SPSB1 (Fig. 4b). TGF-β treatment appeared to have little effect on the SPSB1 degradation rate (Additional file 2: Figure S7).

To further confirm the ubiquitination degradation of SPSB1 by oncogenic Ras, we used proteasome inhibitor MG132 to block the protein degradation. As expected, oncogenic Ras induced protein degradation of SPSB1 slowed by treatment of MG132 (Fig. 4d). Collectively, our results suggest that the oncogenic Ras-SPSB1 interaction results in the enhanced ubiquitination and degradation of SPSB1.

### Ras inhibits SPSB1 mediated ubiquitination of TβRII and hence stabilizes TβRII

**Fig. 5:**
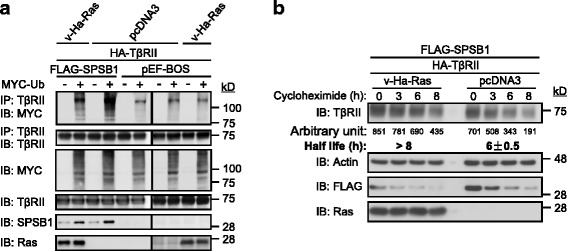
Oncogenic Ras reduces the enhanced ubiquitination of TβRII by SPSB1 and hence stabilizes TβRII. 293 T cells (**a**, **b**) were transfected with indicated FLAG-SPSB1/pEF-BOS control vector together with v-Ha-Ras/pcDNA3 control vector and HA-TβRII ± MYC-ubiquitin. Cells (**a**) were lysed 48 h post-transfection. Cell lysates were immunoprecipitated (IP) with anti-TβRII antibody conjugated with protein G beads. 36 h post-transfection, cells (**b**) were exposed to cycloheximide (20 μg/ml) for indicated periods and lysed. In all cases, both whole cell lysates and immunoprecipatates were examined for indicated proteins by immunoblotting (IB). Relative intensity of each TβRII band (**b**) was qualitatively measured using ImageJ as described in Fig. 4. In all case, results are representative of experiments repeated at least once

Our early data suggest that Ras down-regulates SPSB1 by enhancing its degradation. Since SPSB1 is a negative regulator of TβRII, can Ras regulate TβRII levels via SPSB1 and hence the TGF-β signaling? HA-tagged TβRII [33] was co-transfected with v-Ha-Ras or pcDNA3 vector with or without ubiquitin followed by the immunoprecipitation of TβRII. Consistent with our previous results [31], TβRII was ubiquitinated when it was expressed alone (Fig. 5a). In the presence of oncogenic Ras, we observed no obvious changes in the ubiquitination levels of TβRII (Fig. 5a, last four lanes). As expected, expression of SPSB1 resulted in the increase of ubiquitination levels of TβRII without oncogenic Ras expression (Fig. 5a) [31]. However, co-expression of oncogenic Ras with SPSB1 reduced TβRII ubiquitination levels and SPSB1 expression levels (Fig. 5a). Those data suggest a role of Ras in inhibiting SPSB1’s regulation of TβRII ubiquitination.

Since oncogenic Ras inhibits SPSB1-mediated TβRII ubiquitination, we next investigated the effect of oncogenic Ras on the SPSB1-mediated TβRII degradation using cyclohexmide treatment. As shown in Fig. 5b, in the presence of SPSB1, the half-life of TβRII was measured at ~ 6 h. In contrast, when oncogenic Ras was co-expressed, the degradation rate of TβRII was slowed with its half-life increased to more than 8 h (Fig. 5b). Consistent with those over-expression data, the endogenous TβRI and TβRII levels in the 21D1 cells were observed to be higher than the ones in the partental MDCK cells (Fig. 1a). Collectively, our results suggest that oncogenic Ras compromises the ability of SPSB1-mediated TβRII ubiquitination, and hence, stabilizes TβRII.

### TGF-β receptor levels regulate TGF-β signaling sensitivity and duration

**Fig. 6:**
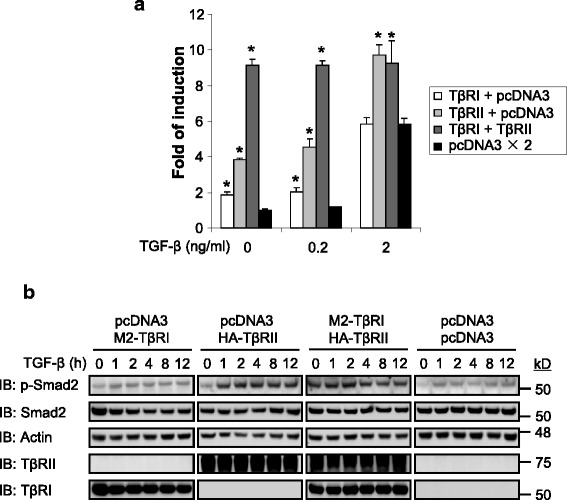
TGF-β receptor levels regulate TGF-β signaling sensitivity and duration. 293 T cells (**a**) were co-transfected with pCAGA-luc and indicated TβRII and/or TβRI and/or pcDNA3 control vector. 24 h later, cells were treated with ± TGF-β at indicated concentration for a further 24 h. Cells were then lysed, and luciferase activity was determined. Data are expressed as relative Smad3 luciferase activity (fold of induction) by standardizing the luciferase activity of un-stimulated cells transfected with contorl vector to 1, and normalizing all other raw values accordingly. Results from a representative experiment are shown as the mean of triplicates±S.D. * *P* < 0.05. 293 T cells (**b**) were co-transfected with indicated DNA constructs. 36 h later, cells were stimulated with ± TGF-β (2 ng/ml) for indicated periods then lysed. Whole cell lysates were examined for indicated proteins by immunoblotting (IB). In all case, each experiment was repeated three times, one representing result is shown

To investigate the effect of TGF-β receptor levels on signaling activity, we used a sensitive TGF-β Smad3 reporter, pCAGA-luciferase. As shown in Fig. 6a, over-expression of TβRI alone in 293 T cells resulted in doubling of basal activity of the luciferase reporter while there was no change of TGF-β (2 ng/ml) stimulated reporter activity in comparison with without any receptor expression. However, over-expression of TβRII alone resulted in even more increase of reporter activity at both basal level (~ 4-fold) and with TGF-β treatments (Fig. 6a). Importantly, over-expression of both TβRI and TβRII elevated significantly the basal reporter activity to similar level as TGF-β treatments (~ 9-fold) (Fig. 6a), indicating an important role of the receptor levels in regulating TGF-β signaling activity. Similar results were also obtained using MDCK cells (Additional file 2: Figure S8).

While pCAGA-luciferase reporter measures Smad3 transcriptional activity, we further investigated the effect of the receptor levels on their another effector Smad2. Without over-expression of either TβRI or TβRII, TGF-β treatment of 293 T cells resulted in phosphorylation of the Smad2 (Fig. 6b). A slight increase of Smad2 phosphorylation levels were observed when TβRI was over-expressed (Fig. 6b). However, over-expression of TβRII alone in 293 T cells resulted in substantial increase of Smad2 phosphorylation levels and the effect lasted 12 h (the maximum duration examined in experiment). Similarly, such increase of Smad2 phosphorylation levels were also obvious when both TβRI and TβRII were over-expressed (Fig. 6b). Those results are consistent with above observations using Smad3 reporter assay, further suggesting the important regulatory role played by the levels of TGF-β receptors in its signaling.

### Ras enhances TGF-β signaling through increase of TβRII levels

**Fig. 7:**
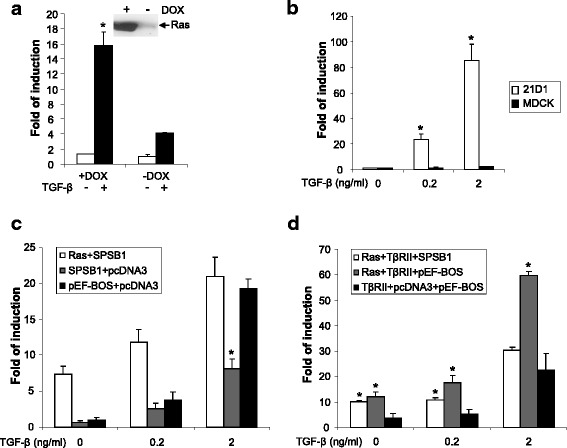
Oncogenic Ras enhances TGF-β signaling. Doxycycline inducible v-Ha-Ras MDCK cells (**a**) were cultured in ± doxycycline (2 μg/ml) for 2 weeks. Cells were then transfected with pCAGA-luc. MDCK and 21D1 cells (**b**) were transfected with pCAGA-luc. 293 T cells (**c**, **d**) were co-transfected with pCAGA-luc and indicated combination of TβRII, TβRI, SPSB1, Ras, pcDNA3 and pEF-BOS constructs. In all cases, 24 h post-transfection, cells were treated with ± TGF-β (2 ng/ml in **a**) at indicated concentration for a further 24 h and lysed. Luciferase activity was determined as desribed in Fig. 6. Data are expressed as mean relative Smad3 luciferase activity (fold-induction) and error bars represent S.D. from representative experiments performed 3 times. * P < 0.05. Western blot of the expression levels of v-Ha-Ras (**a**) was conducted using the same cell lysates for luciferasμe assay. Results are representative of experiments repeated at least once

Since receptor levels regulate TGF-β signlaing activity (Fig. 6) and Ras stablizes receptor levels through destabilization of SPSB1 (Figs. 4 and 5), we further examined the effect of Ras expression on TGF-β signaling. To that end, we generated a doxycycline-inducible Ras expression stable cell line using MDCK cells (Fig. 7a). Indeed, there was a significant increase of TGF-β signaling activity measured by pCAGA-luciferase reporter when the expression of Ras is induced (~ 4-fold) (Fig. 7a). Consistently, TGF-β signaling reporter activity in the Ras transformed 21D1 cells were markedly increased in comparison with parental MDCK cells (~ 32-fold) (Fig. 7b). Those results are in agreement with the observation that Ras transformed 21D1 cells express higher levels of TβRI and TβRII than MDCK cells (Fig. 1a). Conversely, induced expression of SPSB1 (though at low level) in 21D1 cells resulted in reduced TβRII levels (Additional file 2: Figure S9). Consequently, the induction of SPSB1 expression resulted in the reduction of TGF-β reporter activity in 21D1 cells (Additional file 2: Figure S10).

To further investigate the interplay among Ras, SPSB1 and TβRII in TGF-β signaling, we used over-expression and TGF-β-Smad3 reporter assay in 293 T cells. While exprssion of SPSB1 suppressed the reporter activity as expected, however, further over-expression of Ras negated SPSB1’s suppressive effect (Fig. 7c). On the other hand, co-expression of Ras with TβRII markedly increased the reporter activity and that increase was largely suppressed by over-expression of SPSB1 (Fig. 7d).

### Reducing TGF-β signaling in Ras transformed 21D1 cells by SPSB1 suppresses cell migration and invasion

**Fig. 8:**
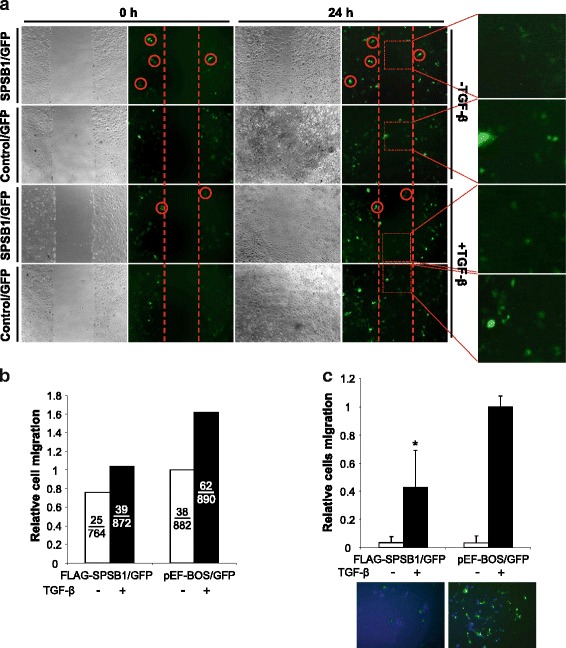
Reducing TGF-β signaling in oncogenic Ras transformed MDCK cells by SPSB1 suppresses cell migration and invasion. 21D1 cells (**a** and **c**) were co-transfected with eGFP construct and FLAG-SPSB1 or pEF-BOS for 48 h. Cell monolayers (**a**) were then scratched as described in materials and methods, treated with ± TGF-β (2 ng/ml) and phase contrast/fluorescence images were recorded at 0 and 24 h post-scratching. Total cell number (denominator) and eGFP expressing cell number (numerator) that migrated into the wounded area were counted and represented as relative cell migration in B. Similar results were obtained in three independent experiments. **c** Following DNA transfection, cell were collected and re-seeded into the upper chamber of 8 μM pore matrigel-coated transwell plates (matrigel 1:1 mixed with DMEM, 70 μl/well) ± TGF-β (2 ng/ml) as indicated for another 24 h. Cells that migrated to the bottom side of the upper chamber were then fixed and stained with Hoechst dye. Images were taken using a fluorescence microscope (magnification = 20×) in 4 random fields. Total cell number (denominator) and eGFP expressing cell number (numerator) that migrated to the bottom side of the upper chamber were counted and represented as relative cell migration. Bars represent the mean ± S.D. of triplicate wells from one of three representative experiments

It has been shown that oncogenic Ras must cooperate with high TGF-β-Smad2 signaling to induce EMT in mouse skin tumour cells [10]. We firstly examined the effect of SPSB1 on the migratory ability of 21D1 cell using wound healing assay. Given the difficulty to express and visualize SPSB1 in 21D1 cells due to the destabilizing effect of Ras, we co-transfected SPSB1 with a eGFP construct to mark SPSB1 expressing cells. Indeed, in 293 T cells, the eGFP expressing cells also expressed SPSB1 (Additional file 2: Figure S11). Since the wild type SPSB1 is not stable in Ras transformed 21D1 cells, we used a non Ras interacting mutant SPSB1(Y129A) to demonstrate the co-expression of eGFP in the same cells (Additional file 2: Figure S12). As shown in Fig. 8a, 21D1 cells almost closed the wounded area in 24 h with or without transfection of SPSB1, while TGF-β treatment resulted in complete closing of the wounded area. Consistently, there were more eGFP labelled cells moved into the wounded area when treated with TGF-β than the cells were not co-transfected with SPBS1 (Fig. 8a & b). However, when cells were co-transfected with SPSB1, non-eGFP labelled cells still moved into the wounded area, but most eGFP labelled cells hardly moved (red circle highlighted cells) and there were apparent reduced numbers of eGFP labelled cells in the wounded area (Fig. 8a & b). While the reduction occured with TGF-β treatment, it also appeared to be ture without TGF-β treatment (Fig. 8a & b).

We then examined the effect of SPSB1 on the invasion property of 21D1 cells using matrigel coated transwell chamber. Again, cells were co-transfected with eGFP construct to mark SPSB1 expressing cells. In the absence of TGF-β, there were hardly any eGFP labelled 21D1 cells on the bottom side of the membrane regardless SPSB1 expression (Fig. 8c). In contrast, with TGF-β treatment but without SPSB1 expression, many eGFP expressing cells moved through the matrigel to the bottom side of the membrane (Fig. 8c). With SPSB1 expression, the number of eGFP labelled cells were reduced by ~ 50% (Fig. 8c). Collectively, those results demonstrate that SPSB1-mediated reduction of TGF-β signaling in Ras transformed 21D1 cells suppresses cell migration and invasion.

## Discussion

TGF-β signaling both suppresses and promotes tumor progression [11]. Functionally, Ras activation overides the cytostatic growth regulation of TGF-β in early tumor development yet synergises with TGF-β signaling to mediate epithelial to mesenchymal transition (EMT) which is the basic cellular process for tumor invasion and metastasis [10, 11, 19]. At molecular level, it has been demonstrated that persistent activation of the MAPK pathway by oncogenic Ras suppresses TGF-β/Smad signaling by inhibiting the nuclear accumulation of R-Smads [24, 43, 44]. Indeed, human colon carcinoma cell lines of known Ras activating mutations show a correlation between the oncogenic Ras and a deficient nuclear accumulation of activated Smad2 and Smad3 [24, 45]. In contrast, Ras signaling has also been shown to up-regulate TGF-β production [27], to enhance endogenous TGF-β signaling [10]. A number of reports have shown that activation of Ras-MAPK pathway can enhance TGF-β-mediated responses in a cell-specific manner. In human mesangial cells, activation of the Erk signaling mediates the TGF-β-induced phosphorylation of Smad3 and leads to the induction of α2(I) collagen promoter activity [46]. Furthermore, in a series of well-characterized tumour cell line derived from sequential stages of mouse skin carcinogensis, activated H-Ras over-expression in squamous carcinoma cells demonstrate that Ras stimulates TGF-β-induced transcription and enhances TGF-β-induced phosphorylated Smad2 levels [10]. However, how Ras positively regulates the TGF-β signaling is not clear. The mechanisms of cross-talk between the Ras and TGF-β signaling are being investigated in a number of cell lines, with controversial results [30].

**Fig. 9:**
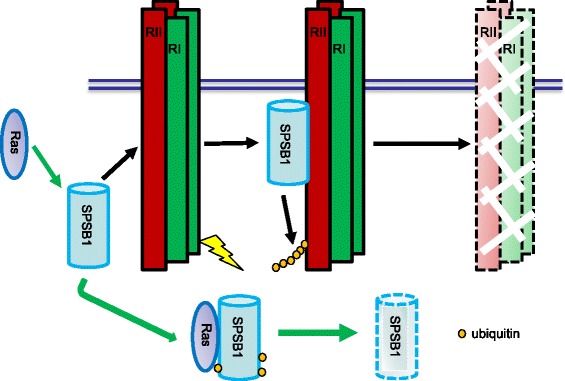
Schematic illustration of Ras enhancing TGF-β signaling through decreasing of SPSB1, a TβRII ubiquitination regulator. SPSB1 interacts with TβRII/TβRI (RII/RI), resulting in polyubiquitination and degradation of the type II receptor (black arrows). Ras interacts with SPSB1, resulting in its protein level decrease through mono-, di-ubiquitination of SPSB1 (green arrows). Consequentluy, Ras enhances TGF-β signaling

Here, we provide the first experimental evidence that Ras can positively regulate the TGF-β signaling by mediating the protein degradation of SPSB1, a newly discovered negative regulator of TβRII. This leads to the stabilization of TβRII. The initial evidence that led to the notion that Ras destabilizes SPSB1 was the difficulty expressing it in Ras transformed 21D1 cells, while its degradation defective mutant SPSB1∆ was readily to be expressed (Fig. 1b). SPSB1 contains a unique SPRY domain which mediates protein-protein interaction [35, 39] and indeed, we identified this domain was involved in its interaction with Ras (Fig. 2). Endogenous Ras interaction with SPSB1 is supported by the observation of a light band (MYC) in lane 5 in Fig. 2b, and to a less extent in the last lane in Fig. 2f. Importantly, a single amino acid mutation (Y129A) in the SPRY domain disrupted not only Ras-SPSB1 interaction (Fig. 2), also negated the ability of Ras to destabilize SPSB1 (Fig. 4c). Using the known SPSB1 recognition motif (D-I-N-N-N-X) [39, 41, 42], we have previously identified a similar motif in TβRII (N^234^-I-N-H-N-T^239^) mediating its interaction with SPSB1 [31]. Use this approach, we identified a short sequence, I^84^-N-N-T-K^88^, in Ras as a candidate SPSB1 interacting motif. However, Ras(N85A) and Ras(N86A) mutants did not disrupt the Ras-SPSB1 interaction (Fig. 2f and Additional file 1: Figure S4), indicating there are other recognition motifs for SPSB1. In searching of them, we aligned the sequences of Ras and c-Met (which also interacts with SPSB1 without containing the D-I-N-N-N-X motif), identifying an identical short sequence (D^120^-L-A-A-R^124^). Interestingly, mutations within this sequence (Ras(D120A, R124A)) resulted in reduced expression level but showed likely enhanced interaction with SPSB1 (Additional file 1: Figure S3 a nd Figs. 5 and 6). The exact motif, which differs from the currently known ones, in mediating Ras-SPSB1 interaction is yet to be identified. Polyubiquitination is the general mechansim leads to protein degradation [42] and polyubiquitination of SPSB1 is observed (Fig. 3a, [31]). Surprisingly, Ras expression had little effect on the polyubiquitination levels of SPSB1 (Fig. 3a), even though Ras has a destabilizing effect on SPSB1 (Fig. 4). Carful analysis of SPSB1 ubiquitination revealed enhancement of mono-, di- and possibly tri-ubiquitination of SPSB1 by Ras (Fig. 3). Whether those mono-, di- and tri-ubiquitination-mediated degradation of SPSB1 is a new mechansim of protein degradation in addition to the normal polyubiquitination requires further investigation. The consequence of Ras-mediated destabilization of SPSB1 is the stabilization of TβRII (Fig. 5). Like some early reports [33], we demonstrated that increased TGF-β receptor levels, particularly TβRII, enhanced TGF-β signaling sentsitivity (Fig. 6). As such, Ras enhances TGF-β signaling through stabilizing TβRII. It is particularly evident in the Ras transformed mesenchymal 21D1 cells that TGF-β signaling is much enhanced in comparison with the parental epithelial MDCK cells through increased TGF-β receptor levels (Figs. 1 and 7). This is the first time a molecular link is demonstrated in which Ras enhances TGF-β signaling (Fig. 9). Furthermore, this Ras-mediated enhancement of TGF-β signaling appears to be responsible for the increased migration and invasion of mesenchymal 21D1 cells since the expression of SPSB1 reduced TGF-β signaling and migration and invasion (Fig. 8). This molecular mechanism underlines the synergistic effect of Ras and TGF-β signaling in mediating EMT.

Recently, SPSB2 has been shown to interact with iNOS and targets iNOS for proteasomal degradation, hence, regulating nitric oxide (NO) production in parasite killing [42]. Soon after, the same group has also demonstrated that SPSB1 is the only SPSB family member to be regulated by the same toll-like receptor (TLR) pathways that induce iNOS expression. And SPSB1 acts through a negative-feedback loop that, together with SPSB2, controls the extent of iNOS induction and NO production [47]. On the other hand, Ras proteins have been shown to positively regulate the nitric oxide synthase family proteins [48, 49]. Our identification of the negative regulatory effect of Ras on SPSB1 may provide a molecular link between the Ras pathway and the nitric oxide synthase pathway.

## Conclusions

We identify Ras as the first negative regulator of SPSB1. In addition, we also uncover a new mechanism of how Ras up-regulates the TGF-β signaling. Ras down-regulates SPSB1 by inducing its protein degradation. This leads to the up-regulation of the TGF-β receptors and consequently, results in the high TGF-β signaling activity (Fig. 9). This is the first report that directly involves Ras protein in the up-regultion of the TGF-β signaling.

## Additional files

Additional file 1: **Figure S1.** EGF stimulation has no effect on SPSB1 protein degradation. 293 T cells were transfected with FLAG-SPSB1. 40 h post-transfection, cells were exposed to cycloheximide (20 μg/ml) for indicated periods with or without EGF (50 μg/ml, 5 mins pretreated) and lysed. Cell lysates were examined for indicated proteins by immunoblotting (IB). Results are representative of experiments repeated at least once. **Figure S2.** EGF stimulation does not alter the interaction between endogenouse Ras and SPSB1. 293 T cells were transfected with FLAG-SPSB1. 48 h post-transfection, indicated cells were stimulated with EGF (50 μg/ml) for 10 min and lysed. Thereafter, cell lysates were immunoprecipitated (IP) with anti-Ras antibody conjugated with protein G beads. Both whole cell lysates and immunoprecipatates were examined for indicated proteins by immunoblotting (IB). Results are representative of experiments repeated at least once. **Figure S3–6.** v-Ha-Ras N85A, v-Ha-Ras N86A and v-Ha-Ras D120A, R124A mutants do not disrupt their ability to interact with SPSB1. 293 T cells (S.3, 4, 5, 6) were transfected with indicated DNA constructs for 48 h. Thereafter, cell lysates were immunoprecipitated (IP) with anti-SPSB1 anibody (S.4) or anti-MYC antibody (S.5) or anti-Ras antibody (S.6) conjugated with protein G beads. Both whole cell lysates and immunoprecipatates were examined for indicated proteins by immunoblotting (IB). In all case, each experiment was repeated at least once, one representing result is shown. (PPT 3970 kb)

Additional file 2: **Figure S7.** v-Ha-Ras N85A and v-Ha-Ras N86A increase the degradation rate of SPSB1. 293 T cells were co-transfected with indicated FLAG-SPSB1 and v-Ha-Ras/v-Ha-Ras mutant/pcDNA3 control vector for 24 h, then cells were treated with TGF-β (2 ng/ml). 36 h post-transfection, cells were exposed to cycloheximide (20 μg/ml) for indicated periods and lysed. Whole cell lysates were then examined for indicated proteins by immunoblotting (IB). Results are representative of experiments repeated at least once. **Figure S8.** TGF-β receptors levels regulate TGF-β signaling sensitivity and duration. MDCK cells were co-transfected with pCAGA-luc and indicated TβRII and/or TβRI and/or pcDNA3 control vector. 24 h later, cells were treated with ± TGF-β at indicated concentration for a further 24 h and lysed. Luciferase activity was determined as desribed in Fig. 6. Data are expressed as mean relative Smad3 luciferase activity (fold-induction) and error bars represent S.D. from representative experiments performed 3 times. * *P* < 0.05. **Figure S9 & 10.** Induced expression of SPSB1 suppresses TGF-β signaling in Ras transformed 21D1 cells through destabilizing TβRII. Doxycycline inducible FLAG-SPSB1 21D1 cells were cultured in ± doxycycline (2 μg/ml) for 2 (S.10) or 7 days (S.9). Whole cell lysates (S.9) were then examined for indicated proteins by immunoblotting (IB). Cells (S.10) were then transfected with pCAGA-luc. 24 h post-transfection, cells were treated with ± TGF-β (0.2 ng/ml) for a further 24 h and lysed. Luciferase activity was determined as desribed in Fig. 6. Data are expressed as mean relative Smad3 luciferase activity (fold-induction) and error bars represent S.D. from representative experiments performed 3 times. * P < 0.05. In all case, each experiment was repeated at least once, one representing result is showing. **Figure S11 & 12.** FLAG-SPSB1 and eGFP co-expression in the cells. 293 T cells (S.11) and 21D1 cells (S.12) were co-transfected with eGFP construct and FLAG-SPSB1/MYC-SPSB1 Y129A as indicated for 48 h. Fixed cells were stained with Hoechst dye. FLAG-SPSB1 was immunostained with mouse anti-FLAG antibody followed by Alexa546-conjugated secondary anti-mouse IgG. MYC-SPSB1 Y129A was immunostained with mouse anti-MYC antibody followed by Alexa546-conjugated secondary anti-mouse IgG. The expression of SPSB1/SPSB1 mutant (red) and eGFP was analyzed by fluorescent microscope (magnification = 20×) in 4 random fields. (PPT 3720 kb)
