# Supplementary material for: Ras enhances TGF-β signaling by decreasing cellular protein levels of its type II receptor negative regulator SPSB1
Source: Cell Commun Signal. 2018 Mar 13;16:10. doi: 10.1186/s12964-018-0223-4 (PMC5850916; doi:10.1186/s12964-018-0223-4)
Supplement: Supplementary file 1 — Figure S1. EGF stimulation has no effect on SPSB1 protein degradation. 293 T cells were transfected with FLAG-SPSB1. 40 h post-transfection, cells were exposed to cycloheximide (20 μg/ml) for indicated periods with or without EGF (50 μg/ml, 5 mins pretreated) and lysed. Cell lysates were examined for indicated proteins by immunoblotting (IB). Results are representative of experiments repeated at least once. Figure S2. EGF stimulation does not alter the interaction between endogenouse Ras and SPSB1. 293 T cells were transfected with FLAG-SPSB1. 48 h post-transfection, indicated cells were stimulated with EGF (50 μg/ml) for 10 min and lysed. Thereafter, cell lysates were immunoprecipitated (IP) with anti-Ras antibody conjugated with protein G beads. Both whole cell lysates and immunoprecipatates were examined for indicated proteins by immunoblotting (IB). Results are representative of experiments repeated at least once. Figure S3–6. v-Ha-Ras N85A, v-Ha-Ras N86A and v-Ha-Ras D120A, R124A mutants do not disrupt their ability to interact with SPSB1. 293 T cells (S.3, 4, 5, 6) were transfected with indicated DNA constructs for 48 h. Thereafter, cell lysates were immunoprecipitated (IP) with anti-SPSB1 anibody (S.4) or anti-MYC antibody (S.5) or anti-Ras antibody (S.6) conjugated with protein G beads. Both whole cell lysates and immunoprecipatates were examined for indicated proteins by immunoblotting (IB). In all case, each experiment was repeated at least once, one representing result is shown. (PPT 3970 kb) [file 12964_2018_223_MOESM1_ESM.ppt]

## Slide 1
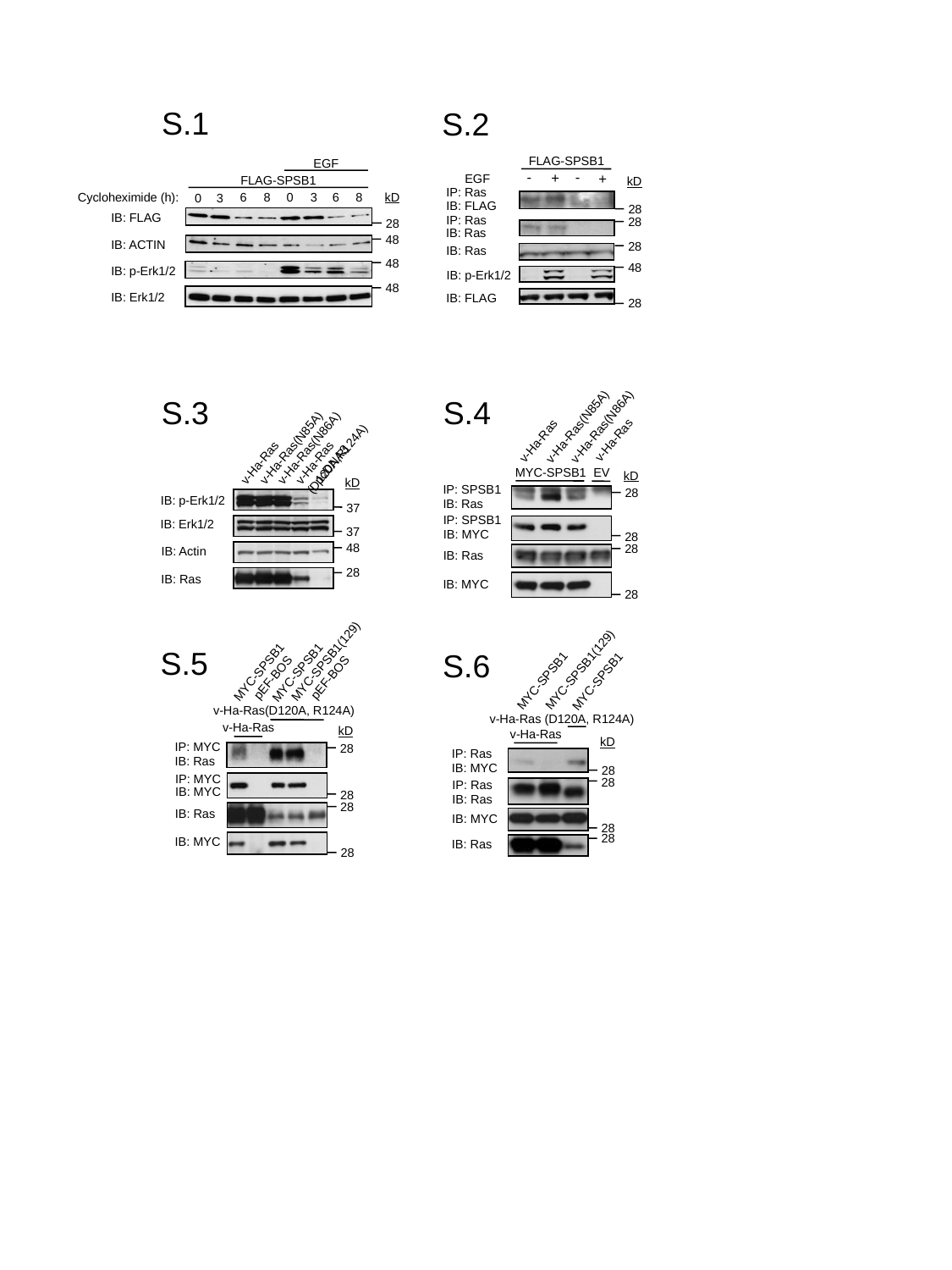

S.1
S.2
FLAG-SPSB1
-
-
+
+
EGF
kD
IP: Ras
IB: FLAG
28
IP: Ras
28
IB: Ras
28
IB: Ras
48
IB: p-Erk1/2
IB: FLAG
28
EGF
FLAG-SPSB1
6
kD
6
0
8
3
8
Cycloheximide (h):
3
0
IB: FLAG
28
48
IB: ACTIN
48
IB: p-Erk1/2
48
IB: Erk1/2
v-Ha-Ras(N85A)
v-Ha-Ras(N86A)
v-Ha-Ras
v-Ha-Ras
MYC-SPSB1
EV
kD
IP: SPSB1
28
IB: Ras
IP: SPSB1
IB: MYC
28
28
IB: Ras
IB: MYC
28
v-Ha-Ras (D120A,R124A)
v-Ha-Ras(N85A)
v-Ha-Ras(N86A)
v-Ha-Ras
pcDNA3
kD
IB: p-Erk1/2
37
IB: Erk1/2
37
48
IB: Actin
28
IB: Ras
S.4
S.3
MYC-SPSB1
MYC-SPSB1
MYC-SPSB1(129)
pEF-BOS
pEF-BOS
v-Ha-Ras(D120A, R124A)
v-Ha-Ras
kD
IP: MYC
28
IB: Ras
IP: MYC
IB: MYC
28
28
IB: Ras
IB: MYC
28
MYC-SPSB1
MYC-SPSB1
MYC-SPSB1(129)
v-Ha-Ras (D120A, R124A)
v-Ha-Ras
kD
IP: Ras
IB: MYC
28
28
IP: Ras
IB: Ras
IB: MYC
28
28
IB: Ras
S.5
S.6
