# Supplementary material for: Ras enhances TGF-β signaling by decreasing cellular protein levels of its type II receptor negative regulator SPSB1
Source: Cell Commun Signal. 2018 Mar 13;16:10. doi: 10.1186/s12964-018-0223-4 (PMC5850916; doi:10.1186/s12964-018-0223-4)
Supplement: Supplementary file 2 — Figure S7. v-Ha-Ras N85A and v-Ha-Ras N86A increase the degradation rate of SPSB1. 293 T cells were co-transfected with indicated FLAG-SPSB1 and v-Ha-Ras/v-Ha-Ras mutant/pcDNA3 control vector for 24 h, then cells were treated with TGF-β (2 ng/ml). 36 h post-transfection, cells were exposed to cycloheximide (20 μg/ml) for indicated periods and lysed. Whole cell lysates were then examined for indicated proteins by immunoblotting (IB). Results are representative of experiments repeated at least once. Figure S8. TGF-β receptors levels regulate TGF-β signaling sensitivity and duration. MDCK cells were co-transfected with pCAGA-luc and indicated TβRII and/or TβRI and/or pcDNA3 control vector. 24 h later, cells were treated with ± TGF-β at indicated concentration for a further 24 h and lysed. Luciferase activity was determined as desribed in Fig. 6. Data are expressed as mean relative Smad3 luciferase activity (fold-induction) and error bars represent S.D. from representative experiments performed 3 times. * P < 0.05. Figure S9 & 10. Induced expression of SPSB1 suppresses TGF-β signaling in Ras transformed 21D1 cells through destabilizing TβRII. Doxycycline inducible FLAG-SPSB1 21D1 cells were cultured in ± doxycycline (2 μg/ml) for 2 (S.10) or 7 days (S.9). Whole cell lysates (S.9) were then examined for indicated proteins by immunoblotting (IB). Cells (S.10) were then transfected with pCAGA-luc. 24 h post-transfection, cells were treated with ± TGF-β (0.2 ng/ml) for a further 24 h and lysed. Luciferase activity was determined as desribed in Fig. 6. Data are expressed as mean relative Smad3 luciferase activity (fold-induction) and error bars represent S.D. from representative experiments performed 3 times. * P < 0.05. In all case, each experiment was repeated at least once, one representing result is showing. Figure S11 & 12. FLAG-SPSB1 and eGFP co-expression in the cells. 293 T cells (S.11) and 21D1 cells (S.12) were co-transfected with eGFP construct and FLAG-SPSB1/MYC- [file 12964_2018_223_MOESM2_ESM.ppt]

## Slide 1
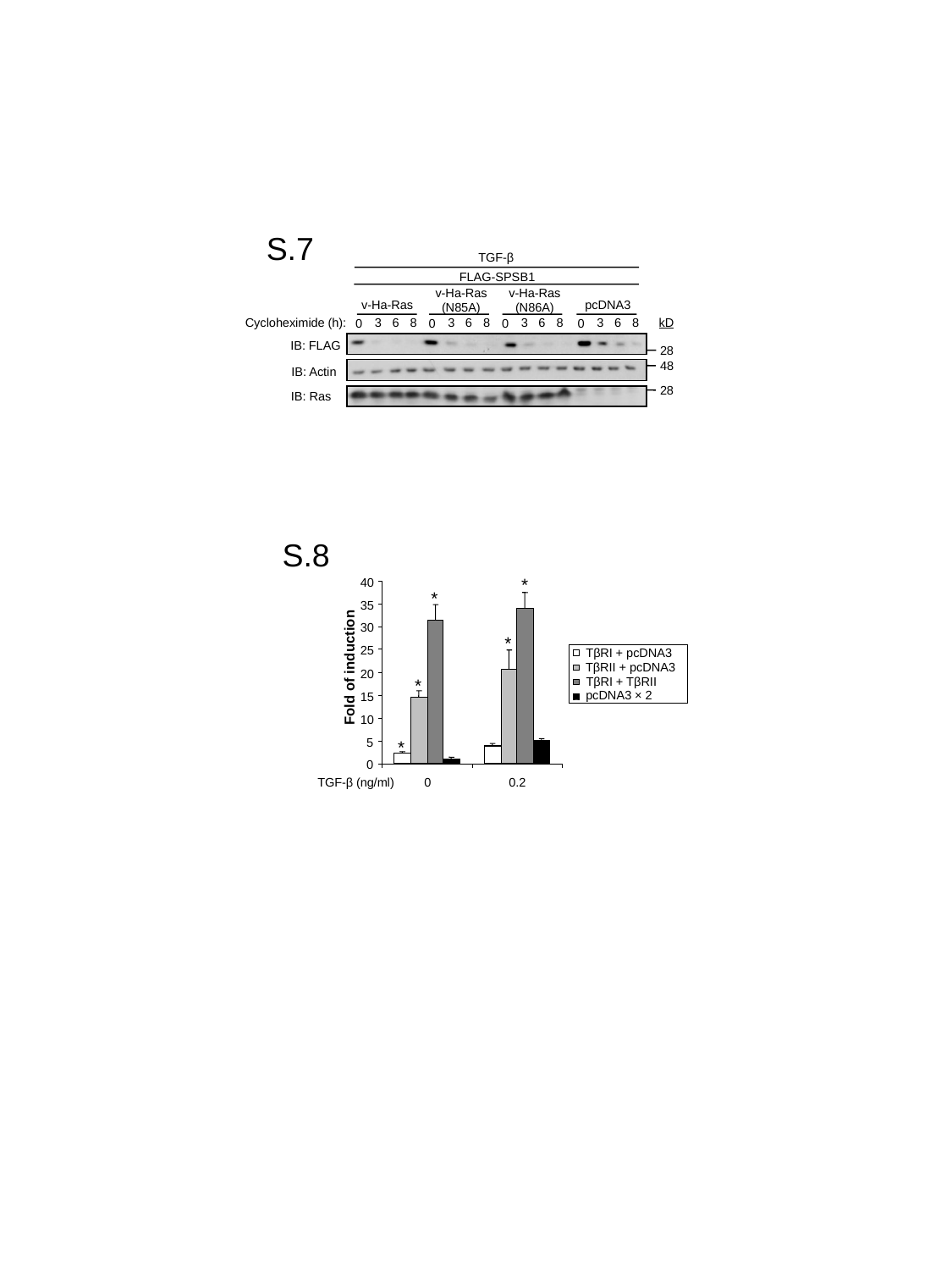

S.7
TGF-β
FLAG-SPSB1
v-Ha-Ras (N85A)
v-Ha-Ras (N86A)
v-Ha-Ras
pcDNA3
Cycloheximide (h):
kD
3
6
8
3
6
8
3
6
8
3
6
8
0
0
0
0
IB: FLAG
28
48
IB: Actin
28
IB: Ras
S.8
*
40
*
35
30
*
25
TβRI + pcDNA3
Fold of induction
TβRII + pcDNA3
20
*
TβRI + TβRII
pcDNA3 × 2
15
10
*
5
0
TGF-β (ng/ml)
0
0.2

## Slide 2
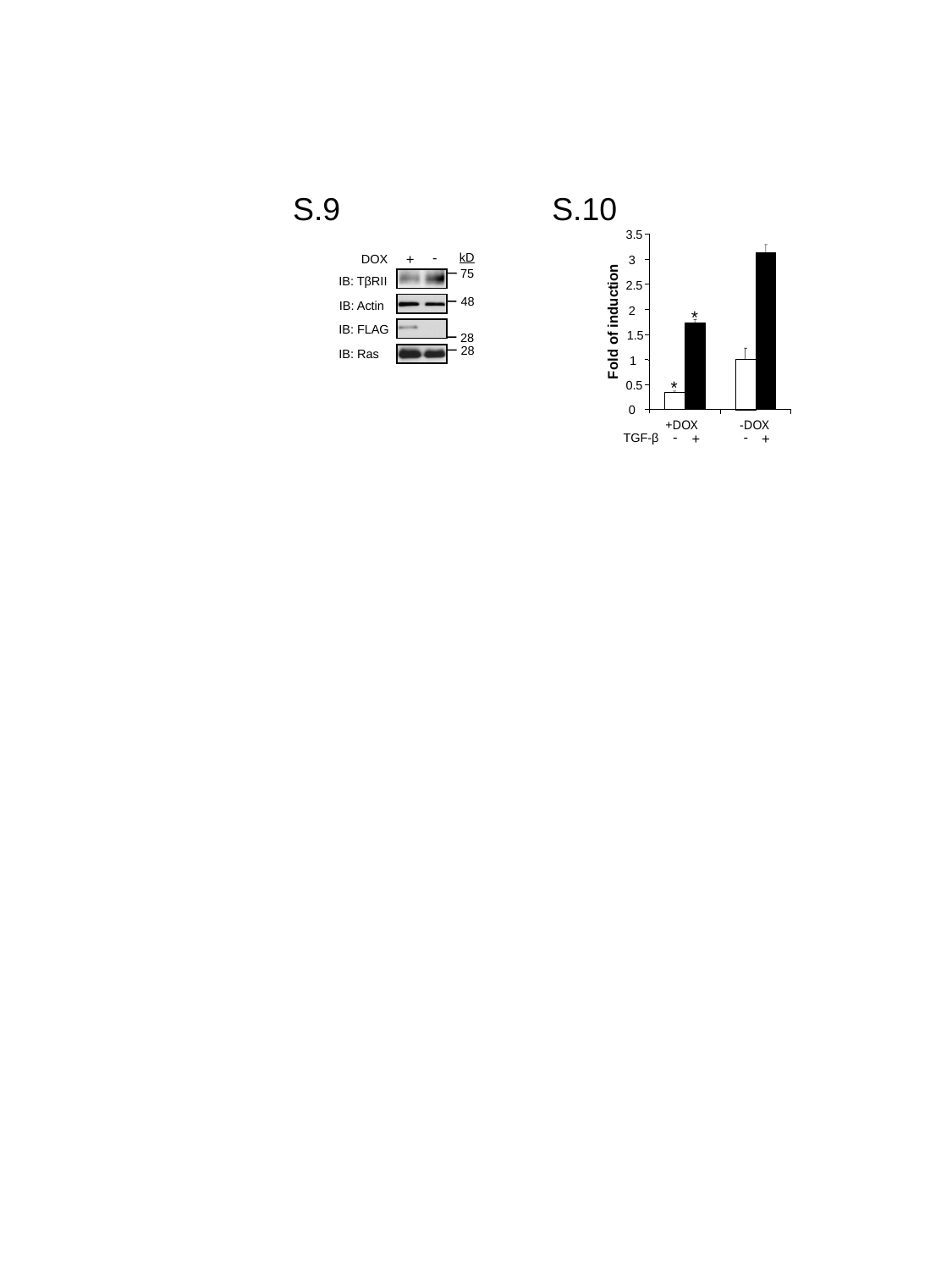

S.9
S.10
3.5
3
2.5
*
2
Fold of induction
1.5
1
*
0.5
0
 +DOX
 -DOX
-
-
+
+
TGF-β
-
kD
+
DOX
75
IB: TβRII
48
IB: Actin
IB: FLAG
28
28
IB: Ras

## Slide 3
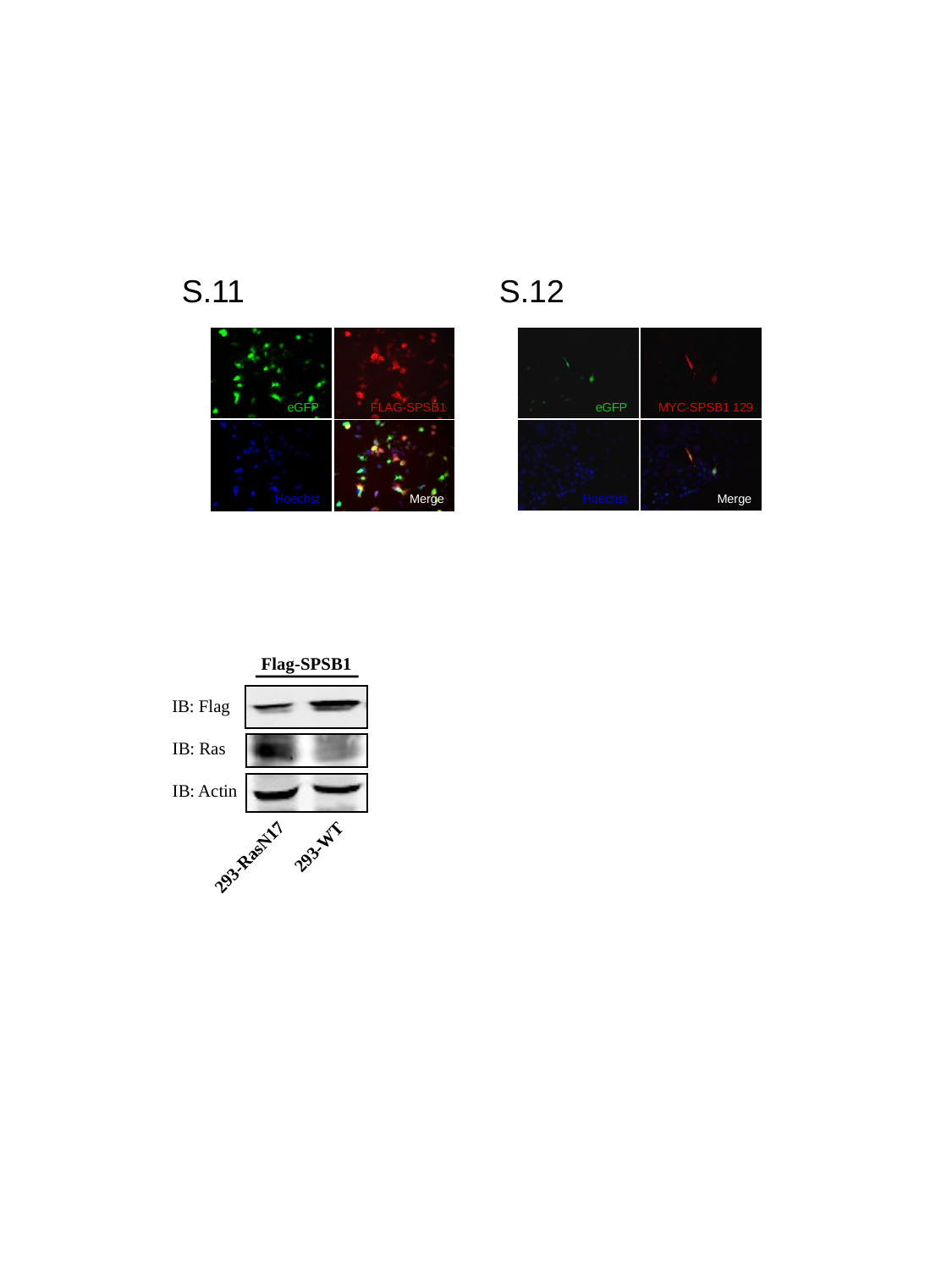

S.11
S.12
FLAG-SPSB1
eGFP
Hoechst
Merge
MYC-SPSB1 129
eGFP
Merge
Hoechst
Flag-SPSB1
IB: Flag
IB: Ras
IB: Actin
293-WT
293-RasN17
